# Assessing HIV-1 subtype C infection dynamics, therapeutic responses and reservoir distribution using a humanized mouse model

**DOI:** 10.3389/fimmu.2025.1552563

**Published:** 2025-04-16

**Authors:** Snehal Kaginkar, Leila Remling-Mulder, Ashashree Sahoo, Tejaswini Pandey, Pranay Gurav, Jyoti Sutar, Amit Kumar Singh, Ella Barnett, Sivasankar Panickan, Ramesh Akkina, Vainav Patel

**Affiliations:** ^1^ Viral Immunopathogenesis Laboratory, Indian Council of Medical Research (ICMR)- National Institute for Research in Reproductive and Child Health, Mumbai, India; ^2^ Department of Microbiology, Immunology and Pathology, Colorado State University, Fort Collins, CO, United States; ^3^ International AIDS Vaccine Initiative (IAVI)- Translational Health Science and Technology Institute (THSTI) Antibody Translational Research Program, Biotechnology Research and Innovation Council (BRIC)-Translational Health Science and Technology Institute, National Capital Region (NCR) Biotech Science Cluster, Faridabad, Haryana, India

**Keywords:** humanized mice for HIV-1C, HIV subtype C infection dynamics in humanized mice, anti-retroviral therapy for HIV-1C, drug resistance mutations, HIV-1C tissue reservoir, treatment interruption

## Abstract

**Introduction:**

While HIV-1 subtype C (HIV-1C) is the most prevalent and widely distributed subtype in the HIV pandemic, nearly all current prevention and therapeutic strategies are based on work with the subtype B (HIV-1B). HIV-1C displays distinct genetic and pathogenic features from that of HIV-1B. Thus, treatment approaches developed for HIV-1B need to be suitably optimized for HIV-1C. A suitable animal model will help delineate comparative aspects of HIV-1C and HIV-1B infections.

**Methods:**

Here, we used a humanized mouse model to evaluate HIV-1C infection, disease progression, response to anti-retroviral therapy (ART) and viral rebound following therapy interruption. A limited comparative study with a prototypical subtype B virus was also performed. Viral infection, immune cell dynamics, acquisition of anti-retroviral therapy (ART) resistance and anatomical reservoir distribution following extended and interrupted therapy were compared.

**Results:**

In comparison, lower early plasma viremia was observed with HIV-1C, but with similar rate of CD4+ T cell depletion as that of HIV-1B. Viral suppression by ART was delayed in the HIV-1C infected group with evidence, in one case, of acquired class wide resistance to integrase inhibitors, a critical component of current global therapy regimens. Also, HIV-1C infected animals displayed faster rebound viremia following ART interruption (ATI). Disparate patterns of tissue proviral DNA distribution were observed following extended ART and ATI suggestive of distinct sources of viral rebound.

**Discussion:**

In this preliminary study, discernible differences were noted between HIV-1C and B with implications for prevention, therapeutics and curative strategies. Results from here also highlight the utility of the hu-HSC mouse model for future expanded studies in this context.

## Introduction

In the ongoing HIV-1 epidemic, HIV-1 subtype C (HIV-1C) accounts for 50.4% of global infections followed by subtype A (12.4%) and subtype B (HIV-1B, 11.3%) (1). HIV-1C has been shown to exhibit unique genomic features that govern co-receptor usage, drug resistance and replication kinetics (2–6). In spite of this subtype being the most prevalent worldwide as well as in low to middle income countries (LMIC), research related to anti-retroviral drugs, broadly neutralizing antibodies, and latency reversal has largely been focused on HIV-1B. To extend promising strategies addressing each of the aforementioned challenges for HIV-1B to HIV-1C, it is thus crucial to model disease progression and response to therapy using an experimental system that is not as resource-intensive as non-human primates and yet able to recapitulate the continuum of disease progression. Also, comparative inter- subtype studies are rare and are prone to confounding by host and environmental factors such as ethnicity of the participants, time of infection and delays in therapy initiation (7). An *in vivo* experimental system that provides a platform to control these variables as well as allows the application of a unified setting to compare subtype C and B viruses with regard to infection kinetics and response to therapy would be ideal. In this context, humanized mouse models with a transplanted human immune system that are permissive to HIV infection offer a suitable avenue (8–12). Indeed, hu-mouse models have been successfully used to study various aspects of HIV infection experimentally. These include studies on viral pathogenesis, viral latency and reversal, mucosal transmission, pre-exposure prophylaxis, anti-retroviral therapies, viral evolution and novel approaches such as gene and cell therapies (13–20). However, all of these studies have utilized subtype B viruses. Here, we employed this model to successfully establish HIV-1C infection and to study disease progression. We then performed a limited comparative assessment of infection and disease progression of HIV-1 subtype B and C viruses, by monitoring plasma viral load, effects on immune cells, accumulation of drug resistance mutations and HIV-1 proviral DNA in different anatomical reservoirs during pre- treatment, on-treatment and treatment interruption phases. Our results showed discernible differences between the C and B subtype viruses.

## Methods

### Cell lines & virus propagation

Representative HIV-1 BaL virus for subtype B and HIV-1 93IN101 for subtype C procured from NIH AIDS reagent programme were used in these comparative studies. SupT1-CCR5+ cells were cultured in RPMI containing 10% FBS and antibiotics. Viruses were cultured and propagated in SupT1-R5 cells. Supernatants were collected during each passage and titred in TZM-bl cells to determine TCID_50_ as previously described (21). D-12 BaL (passage 3) and D-16 93IN101 (passage 4) virus stocks were used to infect mice.

### Generation of hu-mice

Immunodeficient BALB/c Rag1^−/−^γc^−/−^ or BALB/c Rag2^−/−^γc^−/−^ mice were used to generate humanized mice. Briefly, BALB/c is an inbred strain known for its strong Th2 immune responses and susceptibility to tumors and infections. The double mutant immunodeficient BALB/c Rag1 or Rag2^-/-^γc^-/-^ mice lack lymphocytes. This is due to defects in the genes encoding recombinase activating gene (Rag 1 or 2) and common cytokine receptor gamma chain. The Rag mutation prevents normal maturation of T and B lymphocytes by preventing V(D)J recombination. Lack of functional receptors for IL-2, IL-7 and other cytokines inhibits the survival and expansion of lymphocytes, including that of NK cells. The study protocol was approved by the Colorado State University Institutional Animal Center and Use Committee. Briefly, 1 to 3 day old neonatal mice were sub-lethally irradiated at 350 rads and injected intrahepatically with human CD34+ cells (0.5-1 x10^6^ cells/mouse) in a 30 µl volume as previously described (22). Prior to injection, the hCD34+ cells were cultured overnight in Iscove’s media supplemented with 10% FBS and 25 ng/ml of each IL-3, IL-6, Stem Cell Factor (SCF). Ten to twelve weeks post-engraftment, the mice were evaluated for human cell reconstitution. This was done by staining peripheral blood samples with anti hCD45-APC (BD Biosciences; 555485), CD3-FITC (BD Biosciences; 561807) and CD4-PE (BD Biosciences; 555347) antibodies and analyzed by flow cytometry using a BD Accuri cytometer as described (23).

### Infection of hu-mice with HIV-1 subtype C and subtype B virus

Briefly, humanized mice with robust engraftment (>40% hCD45+ cells) (**Supplementary Table 1**, **Supplementary Figure 1**) were intra-peritoneally injected with 50 µl of 10^5.4^ Tissue culture infectious dose_50_ (TCID_50_) of 93IN101 virus (subtype C infected group, HIV-C, n=7; 1 male + 6 female; 18-25 weeks of age). Viral loads were monitored weekly. Approximately 100 µl blood was collected via tail vein with a 50 µl aliquot used for immunostaining and plasma was extracted from the rest. RNA was purified by using E.N.Z.A Viral RNA kit (Omega bio-tek R6874-00). Viral loads were determined by qRT-PCR using iScript™ One-Step RT-PCR Kit With SYBR^®^ Green (Biorad, 170-8892) as per manufacturer’s instructions. CD4+ T cell frequency was evaluated by flow cytometry using antibodies against CD45 FITC (BD, 347463), CD3 APC-Cy7 (BD, 557832), CD4 BV480 (BD, 740161) using a BD Celesta cytometer. Mice similarly infected with 10^5.6^ TCID_50_ of BaL virus (HIV Subtype B) were concurrently analysed. A group injected with PBS only (2 males) was used as uninfected control along with a comparator subtype B infected group, HIV-B (n=7; 3 male+ 4 female; age- 20-27 weeks).

### Anti-retroviral drug administration and analytical treatment interruption

**Figure 1:**
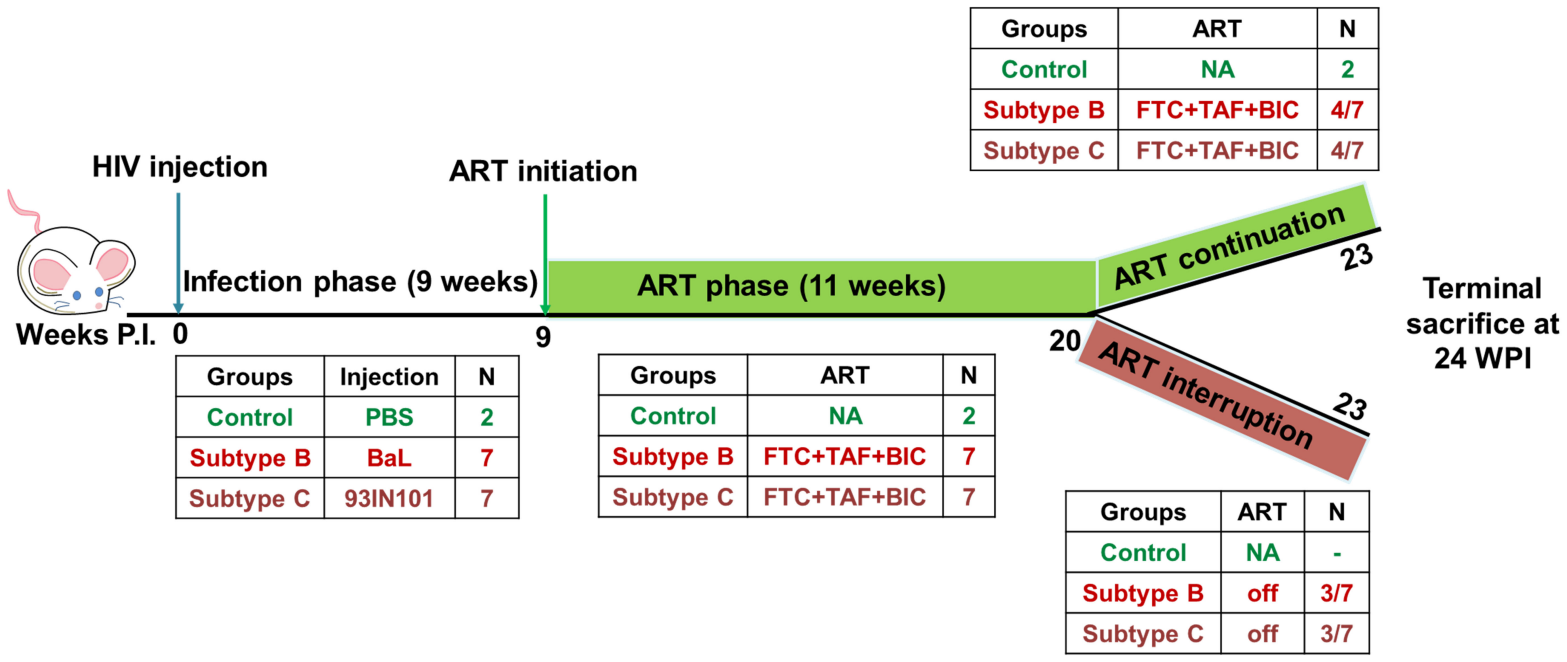
Schematics of experimental design: HIV infection was monitored in hu-mice for a total of 23 weeks. Hu-mice were classified into 3 groups. Uninfected (control, n=2), subtype B infected (HIV-B, n=7), subtype C infected (HIV-C, n=7). The study was divided in 3 phases. In the pre- anti-retroviral therapy phase (ART), up to 9 weeks post infection (WPI) was allowed to occur in the absence of ART. ART phase was initiated at week 9 in viremic mice and continued for 11 weeks i.e. 20 WPI. After ART phase, animals were divided into two study arms. In the ART continuation arm, 4 mice from HIV-B and HIV-C each were continued on ART for a further three weeks. In the second arm, the ART interruption arm, 3 mice from HIV-B and HIV-C each, were released from ART and monitored for viral rebound for 3 weeks. The study was terminated for all animals at 24 WPI.

As depicted in schematic **Figure 1** below, successful establishment of viremia in HIV-B and HIV-C groups was followed by anti-retroviral therapy (ART). Treatment commenced via drug infused food supplement at week 9 post infection, after each infected animal had shown at least 2 consecutive plasma viral loads above the limit of detection. ART was administered for 11 weeks. Anti-retroviral drugs Emtricitabine (Gilead; ICN-019), Tenofovir alafenamide (Gilead; ICN-018) and Bictegravir (Gilead; 102950) were mixed into Dietgel Boost cups (ClearH20, Wesbook, Maine) at a concentration of 82.22 mg/kg, 10 mg/kg, 20.54 mg/kg respectively at 2X the human equivalent dose. Food was changed every alternate day. Blood was collected via tail vein and plasma viral loads were assessed along with immune cell monitoring as described above. At week 20, three mice from each virally suppressed group were then removed from ART for a duration of 3 weeks. At week 24, all mice were sacrificed and their blood and tissues were collected for further analysis.

### Tissue specific HIV proviral DNA distribution

All experimental animals were sacrificed at week 24. Nucleic acids were extracted from bone marrow, mesenteric lymph nodes and blood using TRIzol reagent as per manufacturer’s protocol.

Approximately 100 ng DNA (estimated using 260/280) was subjected to human RPP38 gene specific RT-PCR to estimate copies of human cells (copy number standards were prepared by mixing increasing concentration of J-Lat DNA with CCL-1 mouse cell line DNA). Similarly, 100 ng DNA was subjected to HIV-1 specific *gag* PCR (nested, real time) to estimate copies of HIV DNA (standards were prepared by mixing increasing concentrations of J-Lat DNA with uninfected human PBMC DNA) (24, 25). List of primers is provided in the **Supplementary Table 2**.

### Drug resistance mutations

*Gag-pol* amplicons were generated from plasma HIV RNA samples collected at various time points as previously described (26). Amplicons were prepared for sequencing using QIAseq^®^ FX DNA library kit. NovaSeq 6000 sequencer was used to generate paired end data using PE150 chemistry. Reads were trimmed using Trim galore and checked for their quality. Drug resistance mutations were predicted with the help of Stanford HIVdb DRM prediction system v8.6.1 with a selection criterion of >100 read depth and >1% mutation detection threshold.

### Statistical analysis

Statistical analysis was performed using GraphPad Prism version 8 GraphPad Software, San Diego, California, USA).

Descriptive statistics was used to derive mean and median parameters of the groups. To compare viral load, proviral HIV DNA between the two infected groups we performed non parametric Mann-Whitney test. For CD4+ T cell frequency comparison between control and the two infected groups we used Kruskal- Wallis test by one-way ANOVA. p value less than 0.05 was considered significant.

## Results

### Productive infection of hu-mice by HIV-1C and comparison with HIV-1B

**Figure 2:**
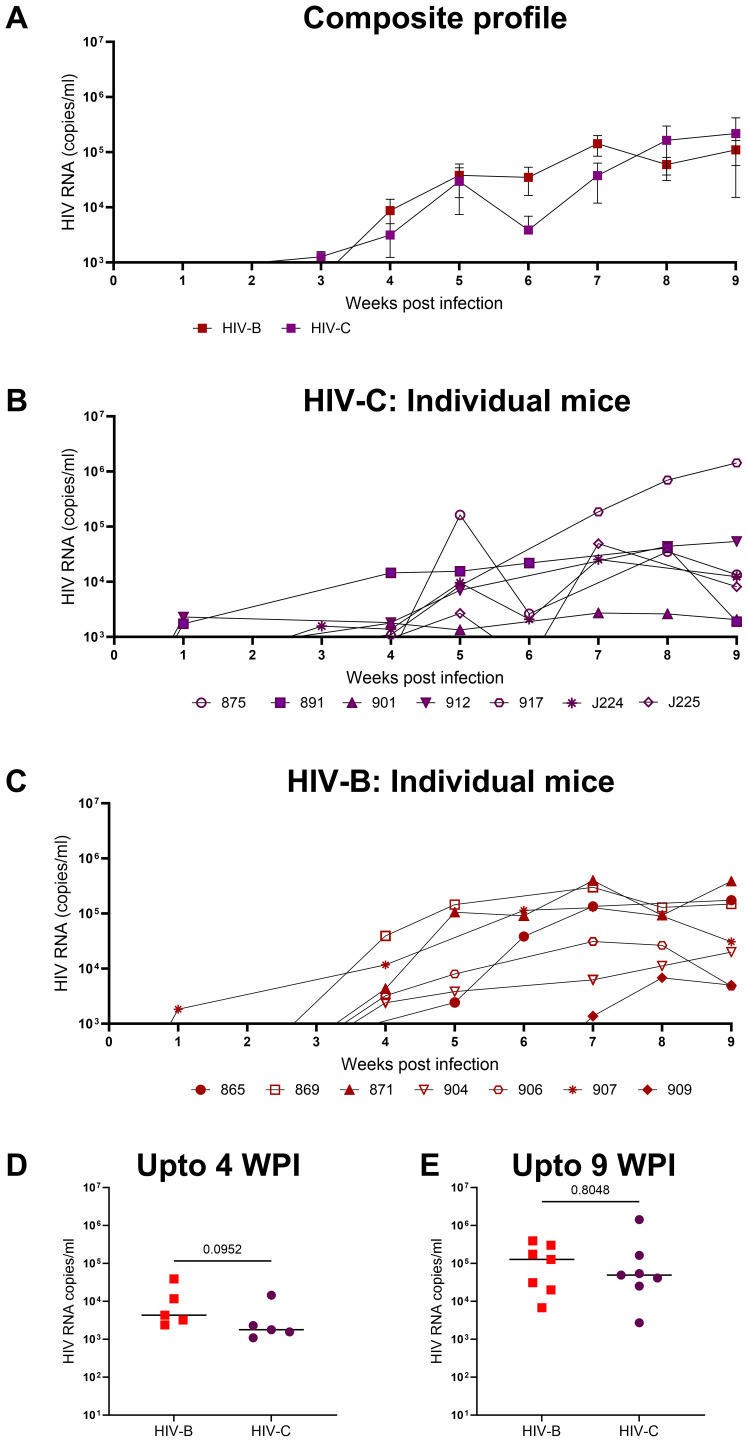
Productive and persistent infection of hu-mice by HIV-1C: Hu-mice were infected i/p with either HIV-1C or HIV-1B and plasma viral loads were assessed by qRT-PCR on a weekly basis. **(A)** Composite weekly viral load profiles of HIV-1C infected group (HIV-C, purple) and HIV-1B infected group (HIV-B, red) shown as mean ± SEM. **(B)** Plasma viremia profile of individual mice from HIV-C group, n=7 and **(C)** individual mice from HIV-B group, n=7. **(D)** Comparison of HIV-C and HIV-B peak viral loads up to 4 WPI and **(E)** from 5-9 WPI respectively done using Mann-Whitney test (p=0.09 and p=0.80 respectively). Horizontal bar shows the median.

In the first set of experiments our goal was to determine if HIV-1C could establish productive infection and sustained viremia in hu-mice. We then compared the data with that of HIV-1B which has been well studied in this system previously. All mice challenged with HIV-1C became viremic similar to those infected with HIV-1B (**Figure 2**). We then compared the results of both HIV-1C infected (HIV-C) and HIV-1B infected (HIV-B) groups in terms of viral loads and infection dynamics as described below. When replication kinetics during this period were compared to those of the HIV-B group (**Figures 2B, C**) we noted that both groups showed a median time point of 4 weeks post infection (WPI) for first detection of viremia (**Supplementary Figure 2B**). Additionally, as evident in **Figure 2D**, when peak viral loads up to 4 WPI were compared, relatively higher (though not significant) viral loads were observed in the HIV-B group (median, 4300 copies/ml) compared to the HIV-C group (median, 1791 copies/ml) during the early period of infection. This trend was maintained during the later phase of infection (up to 9 WPI) (**Figure 2E**). Peak viral loads were attained in both groups with median peak viral loads for HIV-B at 127705 copies/ml and for HIV-C at 49142 copies/ml. All mice remained viremic until the initiation of ART at 9 WPI.

### CD4+ T cell depletion during HIV-C infection

**Figure 3:**
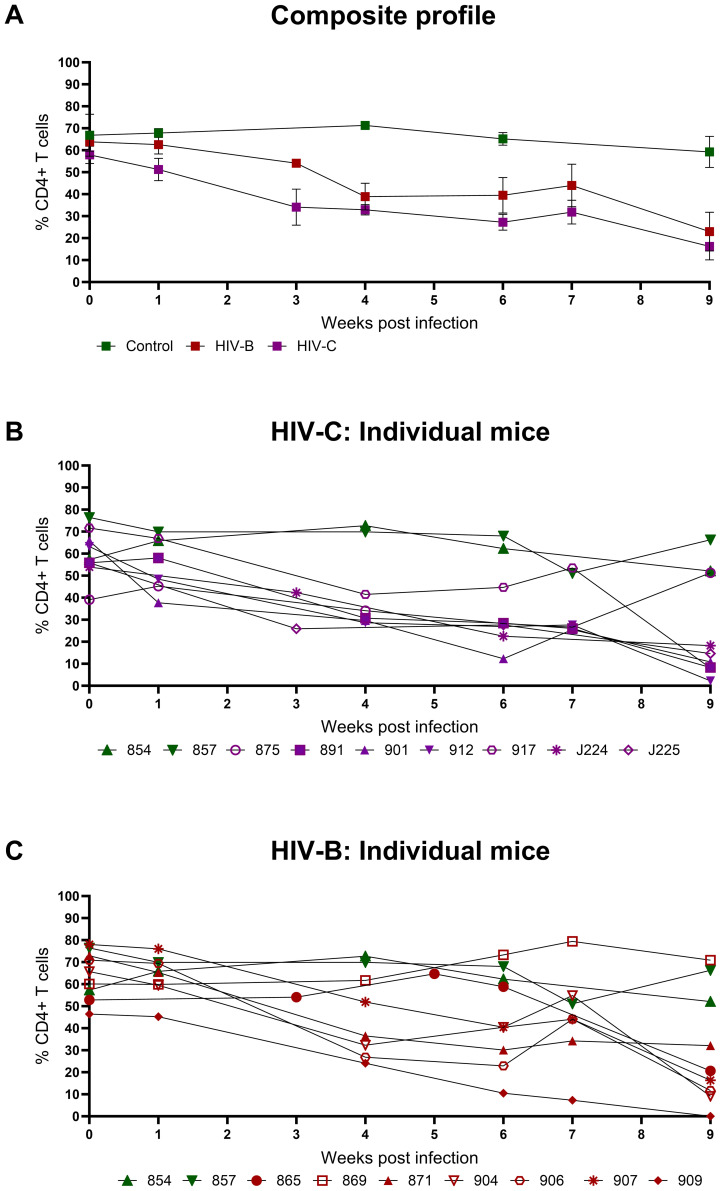
Peripheral CD4+ T cell levels during HIV-1C and HIV-1B infection of hu-mice: Infected mice were bled weekly and CD4+ T cell levels were assayed by flow cytometry. **(A)** Composite weekly CD4+ T cell levels in the HIV-1C infected group (HIV-C, purple) and the HIV-1B infected group (HIV-B, red) along with the uninfected group (control, green), shown as mean ± SEM were compared using the Kruskal- Wallis test **(B)** CD4+ T cell levels of individual mice from the HIV-C group (n=7) and **(C)** Individual mice from the HIV-B group, (n=7) along with control animals (n=2).

Since CD4+ T cell depletion is a hallmark of HIV infection, this was evaluated in infected mice. As shown in **Figures 3A–C** and **Table 1**, all infected mice (except one in HIV-B), exhibited CD4+ T cells depletion post infection with variable nadir levels and duration compared to the controls. Median of nadir CD4+ T cells frequency in HIV-B infected mice was 14.05% and in HIV-C was 10.8% which was not significantly different (**Supplementary Figure 2C**, p=0.62). The median time of the lowest CD4+ T cells frequencies in both groups was observed 9 WPI (**Supplementary Figure 2D**). We observed similar CD4+ T cell % depletion in HIV-B and HIV-C groups (**Table 1**). Although, as shown earlier (**Figure 2E**), viremia trended higher in HIV-B. Interestingly however, the HIV-B animal (No. 869, **Figure 3C**) not showing evidence of depletion had high levels of initial (at 4 WPI) and peak (at 7 WPI) viremia shown above in **Figure 2C**.

**Table 1:** Pathogenesis parameters during the study.

Infection phase (0-9 WPI)	Groups		ART phase (9-20 WPI)	Groups		ATI phase (20-23 WPI)	Groups	
	HIV-B	HIV-C		HIV-B	HIV-C		HIV-B	HIV-C
Viremia^#^ Copies/ml	(7/7), 127705	(7/7), 49142	Viremia^#^ Copies/ml	(7/7), TND	(6/7), TND	Viremia^#^ Copies/ml	(2/3), 5224	(3/3), 2779
% CD4+ T cell depletion ^a^	(6/7), 81	(7/7), 83	Fold CD4+ T cell expansion ^b^	(6/7), 4.2	(7/7), 6.1	% CD4+ T cell depletion ^a^	(3/3), 30	(2/3), 43

Number of animals in each group showing the respective phenotype are indicated in parentheses with the corresponding median value for the group provided after the comma.

^#^Viremia during infection phase and ATI phases represents the peak viremia and during the ART phase is the nadir value.

^a^% reduction of CD4+ T cells was calculated by subtracting nadir frequency from that at 0 WPI for the infection phase. For the ATI phase % reduction was calculated by subtracting the nadir value of ATI phase from the peak value in the ART phase.

^b^Fold increase in CD4+ T cell frequency in the ART phase was calculated by dividing the peak value with the nadir value in the infection phase.

### Response to ART and viral rebound kinetics upon treatment interruption

**Figure 4:**
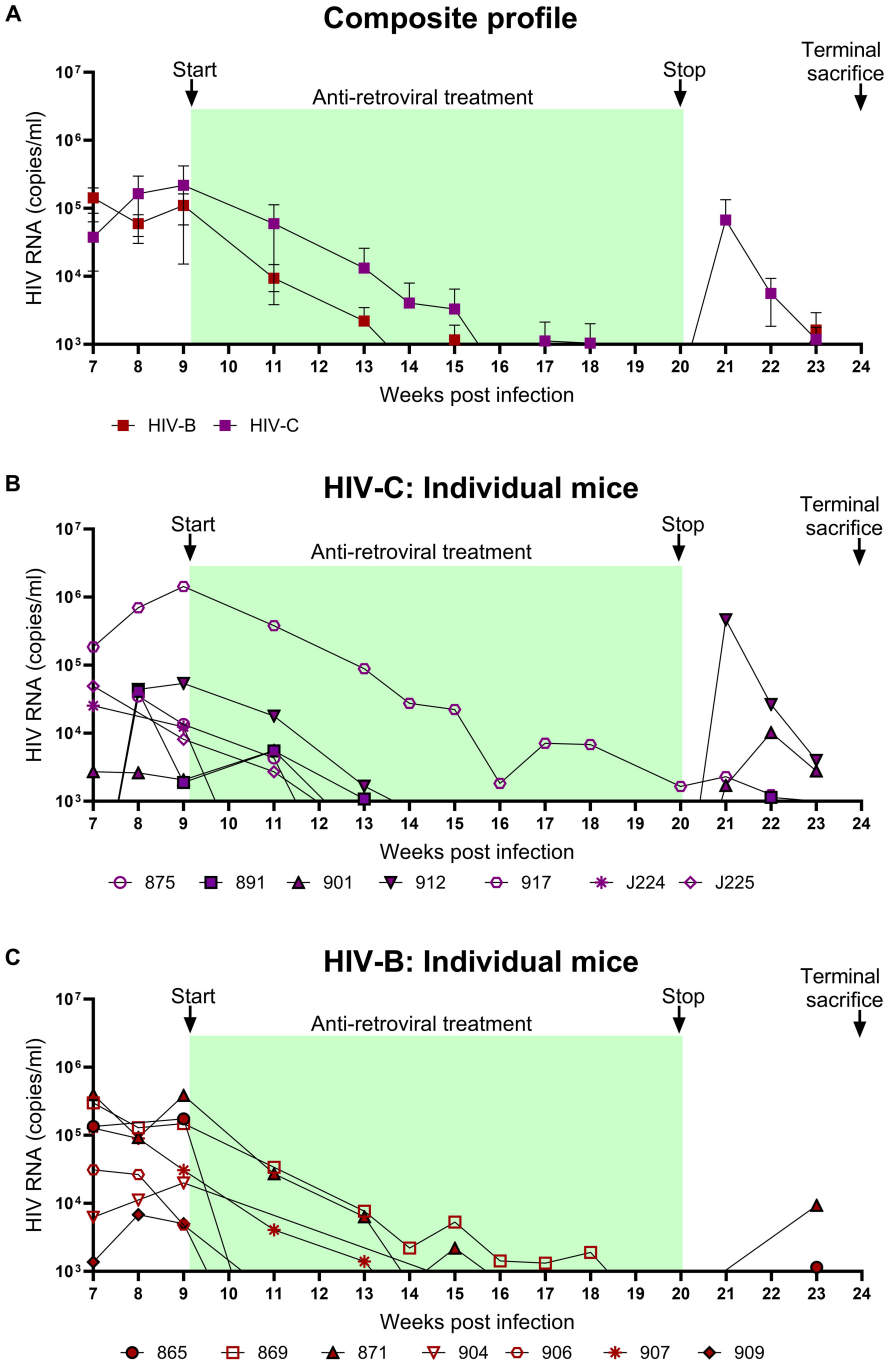
Viral suppression during ART and viral rebound kinetics following treatment interruption: After confirming chronic viremia, ART was commenced at week 9 and continued for 11 weeks. For treatment interruption, ART was discontinued in 3 mice from each group for 3 weeks prior to sacrifice of all mice. Plasma viral loads were determined by qRT-PCR on a weekly basis. **(A)** Composite weekly viral load profiles of HIV-C (purple) and HIV-B (red) infected groups shown as mean ± SEM. **(B)** Plasma viremia profile of individual mice from the HIV-C group (n=7) and **(C)** individual mice from HIV-B group (n=7). Green shaded areas represent duration of ART treatment. At 20 WPI, 3 mice from each of the HIV-B and HIV-C groups were released from therapy (represented as closed symbols with black border) and their viral loads were monitored until 23 WPI. Study was terminated with sacrifice at 24 WPI of all animals, including those continued on ART from 9 WPI.

To evaluate the utility of hu-mice in determining the efficacy of combinatorial ART against HIV-1C, treatment was initiated 9 WPI. HIV-1B infected mice were treated similarly. As shown in **Figure 4**, both groups showed viral suppression as expected. However, some differences in viral suppression kinetics were noted between HIV-C and HIV-B groups. Interestingly (**Figures 4B, C**), in spite of similar viral loads at 9 WPI, plasma viremia remained detectable in only 3/7 mice in HIV-B but 6/7 mice in HIV-C after 11 WPI (2 weeks post ART initiation). Complete virological suppression could not be achieved in the HIV-C group, even by 14 WPI (5-week post ART initiation), with one animal remaining viremic during the entire ART phase which extended to 20 WPI (**Figure 4B**, No. 917). In contrast, 6/7 HIV-B animals were completely suppressed by 14 WPI with one mouse demonstrating a viral blip at 15 WPI. Thereafter, all mice (except animal 869) showed complete suppression by 18 WPI. Additionally, we compared plasma viremia in HIV-B and HIV-C groups at each time point (**Figure 4A**) and did not observe significant differences in viral loads (when detectable) between groups (**Supplementary Figure 3**, **Table 1**). To determine viral rebound kinetics following treatment interruption, ART was interrupted in 3 virologically suppressed animals from both HIV-B and HIV-C groups at 20 WPI (**Figures 4B, C**). All three treatment released HIV-C animals showed detectable viremia by 22 WPI (i.e. 2 weeks post interruption), indeed, 2 of these animals showed detectable viremia at 21 WPI (1 week post interruption, **Figure 4B**). In contrast, only 2 HIV-B animals showed detectable viremia at week 23, suggestive of a slower viral rebound following treatment interruption (**Figure 4C**). Post treatment interruption, viral load rebound was evaluated for 3 weeks until 23 WPI before terminal sacrifice at 24 WPI.

### CD4+ T cell dynamics during ART and treatment interruption

**Figure 5:**
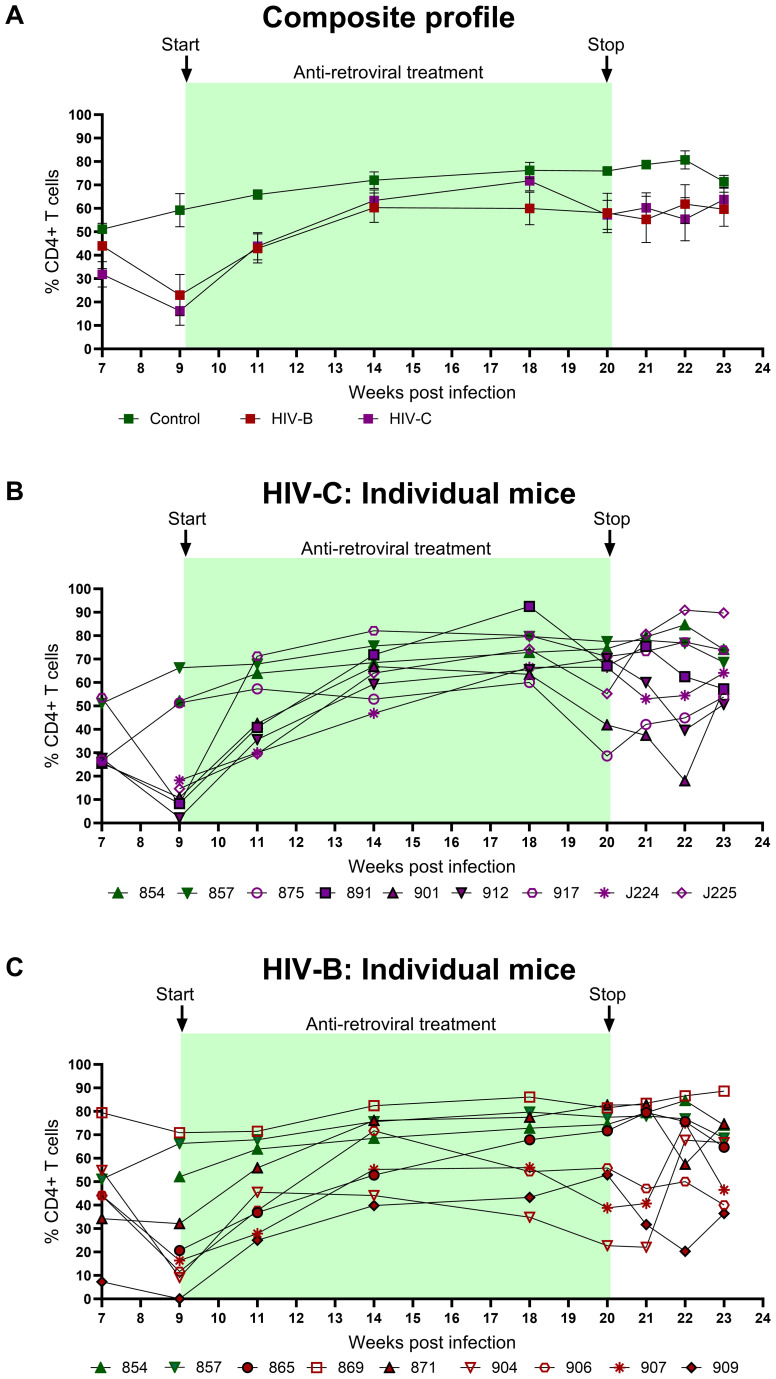
Peripheral CD4+ T cell profiles during ART and treatment interruption: CD4+ T cell levels were assessed by flow cytometry on a weekly basis. **(A)** Composite CD4+ T cell profiles of uninfected control (green), HIV-B group (red) and HIV-C group (purple). Intergroup comparison was done using the Kruskal- Wallis test. **(B)** Frequency of CD4+ T cells in individual mice from HIV-C group (n=7) and **(C)** individual mice HIV-B group (n=7) along with control animals.

CD4+ T cell frequency was monitored in all groups concurrently (**Figures 5A–C**) to evaluate potential recovery during ART in HIV-B and HIV-C groups. Rebound in CD4+ T cell frequency was observed in all HIV-C animals that had also shown CD4+ T cell depletion in the infection phase (**Figure 5B**). Interestingly, animal 917, which had remained viremic during ART also demonstrated rebound. Similar rebound profiles of CD4+ T cell frequency were observed in 6/7 animals in HIV-B group, excluding animal 869, (**Figure 5C**, **Table 1**), which had not shown depletion during the infection phase (**Figure 3C**). During treatment interruption, all 3 ART interrupted HIV-C animals reverted to showing CD4+ T cell depletion in at least 2 out of 3 time points sampled (**Figure 5B**). In the case of 3 treatment interrupted HIV-B mice, CD4+ T cell depletion was observed in all animals for at least one time point before study termination at 24 WPI. Interestingly, HIV-B animal 909 that did not exhibit rebound viremia (**Figure 4C**) showed evidence of CD4+ T cell depletion.

### Assessment of drug-resistant mutations

**Figure 6:**
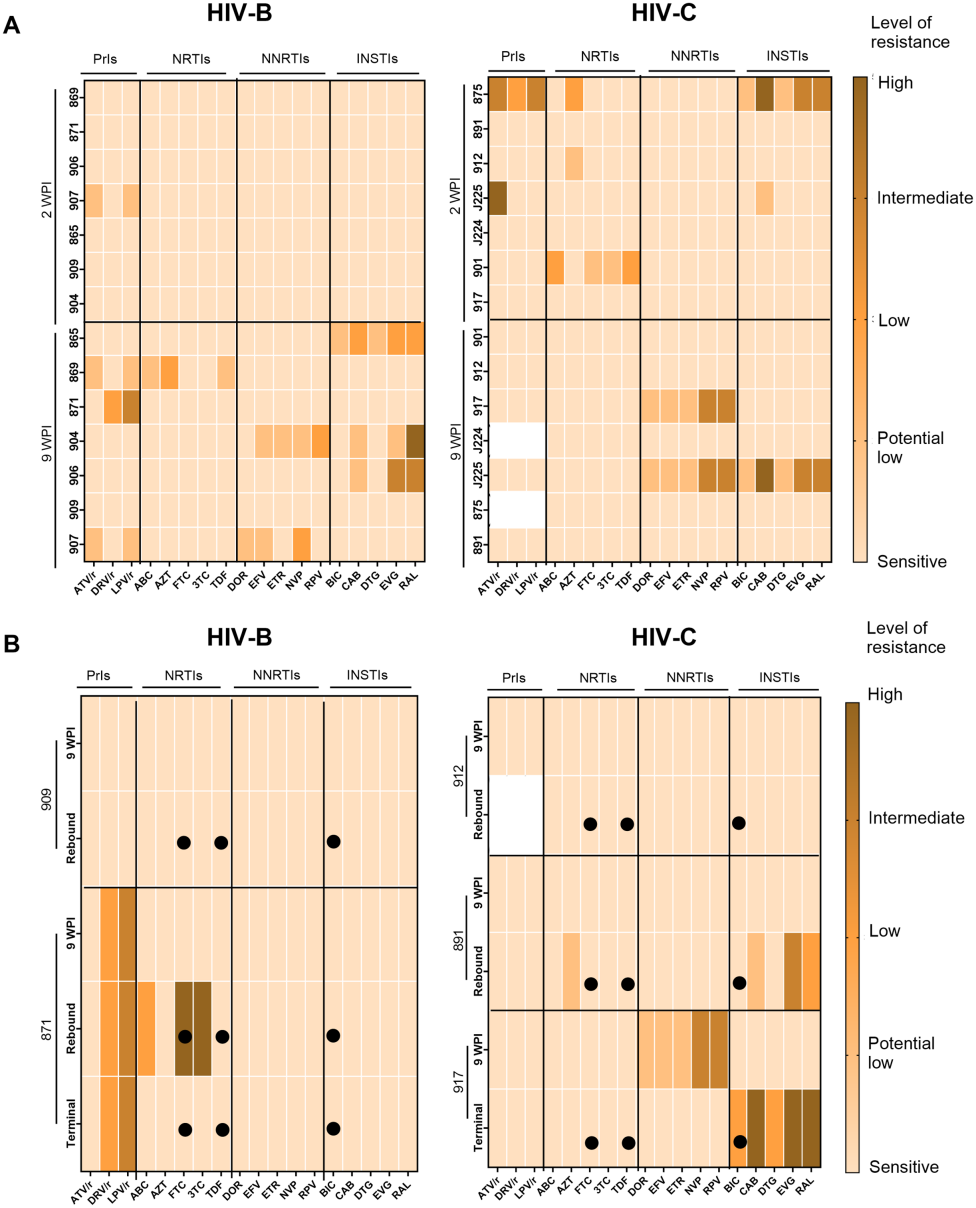
Assessment of drug resistance mutations in circulating viruses during pre-treatment and post-ART. Colour key indicates level of resistance with lighter to darker shades representing sensitive (no resistance), potential low level, low level, intermediate level and high level of resistance. **(A)** Heatmap of *pol* sequences indicating presence and level of DRM conferred resistance to classes of antiretroviral Protease Inhibitors (PrIs), Nucleoside Reverse Transcriptase inhibitors (NRTIs), Non-Nucleotide Reverse Transcriptase Inhibitors (NNRTIs) and Integrase Strand Transfer Inhibitors (INSTIs) with drugs on the X axis and animal IDs on the Y axis during infection phase **(B)** Heatmaps of longitudinally sampled animals from HIV-B (909 and 871) and HIV-C (912 and 891) groups in treatment release arm, and HIV-C animal 917 that received uninterrupted therapy. For each animal, ART drugs are listed on the X-axis and time points for DRM analysis are shown on the Y-axis along with animal IDs. Solid circles indicate the drugs administered during therapy.

As described above, our observations of apparently distinct ART mediated viral suppression and rebound (upon treatment interruption) profiles across HIV-B and HIV-C groups led us to evaluate if distinct accrual of drug resistance mutations (DRM) also occurred. Thus, we evaluated DRM profiles across the different phases of infection, therapy and treatment interruption (**Figure 6**). At 2 WPI, the HIV-C group showed greater prevalence of DRMs in all drug classes except Non-Nucleoside Reverse Transcriptase Inhibitors (NNRTIs). Whereas, in the later phase of infection i.e. 9 WPI, DRMs were observed across classes in both groups (**Figure 6A**). In a subset of animals from each group we obtained longitudinal data from treatment interruption and study termination time points shown in **Figure 6B**. With respect to treatment interrupted animals, mouse 909 (HIV-B) and 912 (HIV-C) did not show presence of DRMs in their longitudinal samples (**Figure 6B**). Animal 871 (HIV-B) consistently had Protease inhibitors (PrIs) mutations at all the phases with acquisition of Nucleoside Reverse Transcriptase Inhibitors (NRTIs) mutations in rebound viremia. Further, HIV-C mouse 891 showed DRMs against NRTIs and Integrase Strand Transfer Inhibitors (INSTIs) in rebound viremia. Notably, HIV-C animal 917, that never achieved undetectable viremia following ART (**Figure 4B**), displayed distinct profiles in infection and terminal time points wherein the latter included DRMs specific to BIC.

### HIV-1 reservoir status

**Figure 7:**
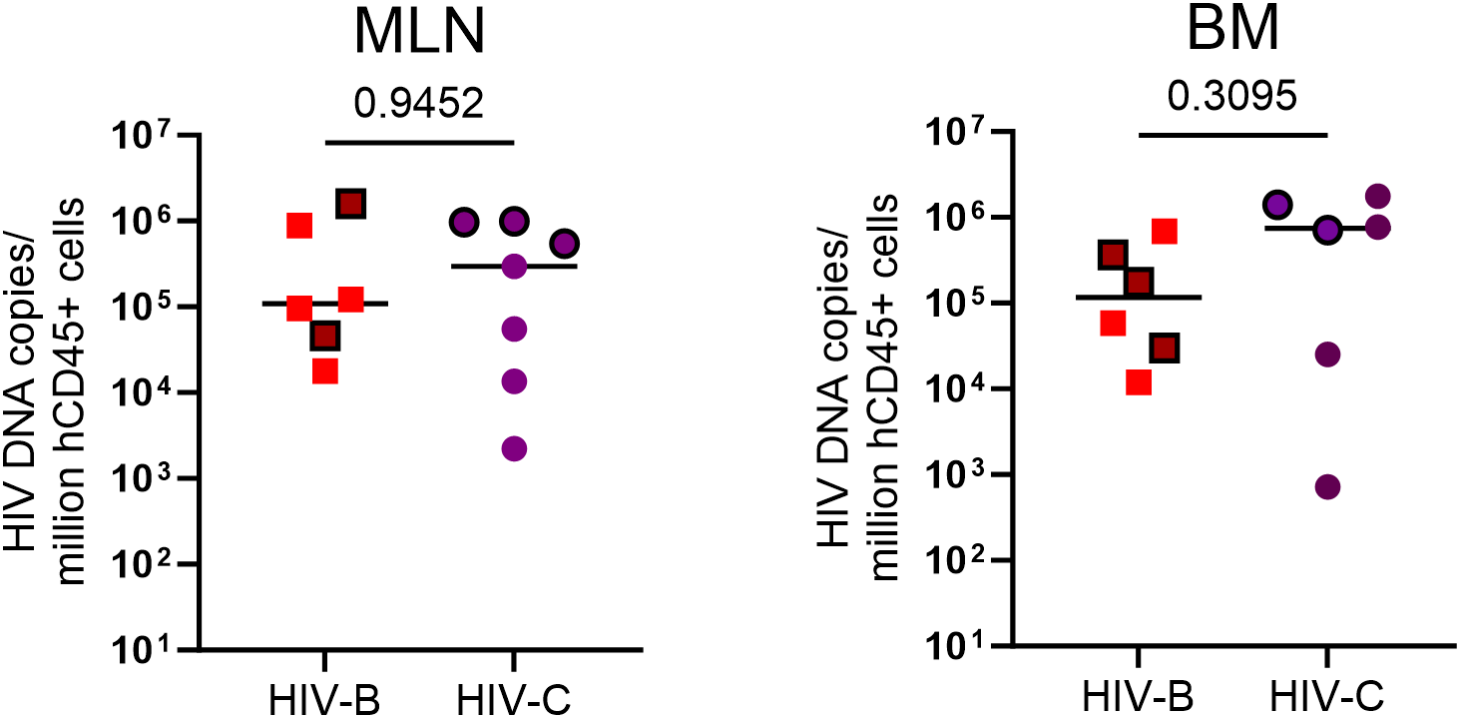
HIV proviral DNA distribution in tissues: Terminal tissue collection samples were analyzed by PCR to measure proviral loads. From left to right, HIV proviral burden in mesenteric lymph nodes (MLN) and bone marrow (BM). Values expressed as proviral copies per million hCD45+ cells. HIV DNA proviral copies were compared using the Mann-Whitney test group wise comparison of all animals. Horizontal bar shows the median. Symbols with black borders indicate treatment interrupted animals in both the groups.

Since viral loads could be influenced by reservoir size and its distribution in tissues at different sites, we sought to assess proviral loads when possible, across 2 anatomical sites at study termination. At terminal sacrifice (24 WPI), as shown in **Figure 7** and **Supplementary Figure 4**, tissues (mesenteric lymph node, MLN; bone marrow, BM) were harvested and HIV proviral DNA load was estimated. Proviral burden trended higher in HIV-C (**Figure 7**) animals overall, across tissues. Stratification of animals into ART continuation and ATI arms (**Table 2**, **Supplementary Figure 4**) showed indications of inter-subtype differences. In the ART continuation arm, HIV-B MLN had the highest median proviral load (MPV) followed by BM. In HIV-C, BM had the highest MPV followed by MLN. Proviral burden in HIV-B animals, compared to that in HIV-C group, was higher in MLN. Conversely, BM proviral load was observed to be higher in HIV-C animals. In the ATI arm, MPV increased in all tissues. Also, the hierarchy of MPV across tissues remained the same as observed in the treatment continuation arm. Further, fold increases in MPV was similar for HIV-B and HIV-C in BM. Strikingly, MPV was higher in MLN for HIV-C animals in contrast to the HIV-B group. Interestingly, fold increases in MPV suggested that the source of rebound viremia for both groups was MLN, in spite of BM exhibiting the highest MPV for HIV-C animals in the ART continuation arm.

**Table 2:** Putative HIV-1 reservoir distribution in HIV-B and HIV-C groups.

	ART continuation (9-24 WPI)		ART interruption (20-24 WPI)		Fold change in median proviral loads	
	HIV-B	HIV-C	HIV-B	HIV-C	HIV-B	HIV-C
MLN	109392, n=4	34365, n=4	818449, n=2	970357, n=3	7.48	28.23
BM	58523, n=3	399217, n=4	174929, n=3	1059386, n=2	2.98	2.65

Median proviral loads (MPV) across tissue compartments (MLN, mesenteric lymph nodes and BM, bone marrow) is shown for each group. Fold change in MPV was calculated by dividing MPV of ART interruption arm with that of ART continuation arm.

## Discussion

Despite its wide prevalence both in terms of geographic distribution and in number of people infected, controlled experimental studies on HIV-1C in an animal model have been lacking to date. To our knowledge, this is the first description of an integrated approach describing *in vivo* infection dynamics of HIV-1C virus, its response to anti-retroviral therapy (ART) and treatment interruption using humanized mouse model. Previous data derived from human clinical studies on both HIV-1C and HIV-1B viruses suggested some notable differences in replication capacity and pathogenesis, but many of these were confounded by variables involving host factors, duration of untreated infection and that of therapy adherence thus obscuring the true picture of inherent differences between these subtypes. In this study we report on viral kinetics, comparative pathogenesis, and reservoir burdens for the globally prevalent HIV-1C subtype in relation to the most widely studied HIV-1B using the hu-mouse *in vivo* model wherein the study conditions were uniform and could be controlled. Our initial experiments focused on establishing HIV-1C infection in hu-mice and comparing the data to that of HIV-1B virus. Our results showed that viral infection could be readily established with the HIV-C, similar to that of HIV-B. However, the median viral loads during the initial four weeks were lower for HIV-C compared to HIV-B. A previous study of a shorter duration (8-10 weeks) demonstrated infection of hu-mice with a different set of viral strains, namely ADA (subtype B) and C_1157_ (African subtype C) (27). While 100% productive infection and CD4+ T cell decline was seen with the ADA strain, only 50% infection and no CD4+ T cell decline was noted with the C_1157_ virus. This is in contrast to our results wherein all mice showed productive infection and CD4+ T cell decline with the HIV-1C 93IN101 virus. This could be due to either differences in strain C viruses used and/or lower infecting dose TCID_50_ (10^4^) compared to that used in our study (10^5^ each of BaL and 93IN101). Overall, the above results showed permissiveness of hu-mouse model to infection with African and Indian strains of HIV-1C.

The lower viremia observed in HIV-C group at week 4 (early infection stage) could be due to a lower number of initially infected cells. This observation may reflect distinct genetic profiles in viral genes governing fusion, cell to cell transmission efficiency (2, 28) or RT and Pro activity (29, 30). We next analyzed and noted a clear trend in CD4+ T cell decline similar to that seen within HIV-B.

In human studies, plasma viremia in HIV-1C individuals has been reported to be overall lower, though not drastically, compared to subtypes A, B, D at the time of diagnosis in patients of diverse ethnicities. Moreover, this study reported similar rates of CD4+ T cell decline across HIV-1B and HIV-1C infections (31). Despite the early differences in viremia observed in HIV-C animals within our study, pathogenic profiles in terms of CD4+ T cell depletion remained comparable. Thus, our results demonstrate the utility of hu-mice in comparing viral growth kinetics and CD4+ T cell decline between subtypes without the confounding factors inherent in previous human clinical studies. Interestingly, a single mouse in HIV-B group did not show evidence of CD4+ T cell depletion in spite of high early and late viremia suggestive of viremic non-progressor phenotype identified by us and others (32, 33). However, the scope of this study could not address this issue and it can be further investigated in other studies where long-term untreated infections are followed.

Having established productive and persistent viremia with HIV-1C virus in hu-mice, we next proceeded to evaluate the response to an ART regimen comprising of BIC/FTC/TAF. Our results showed that HIV-C group responded to ART similar to the HIV-B animals, albeit with some differences.

Following ART initiation, mice with higher initial viral loads (>10^5^ copies/ml) took longer to achieve viral suppression consistent with human clinical data showing pre-ART viral load was a predictor for time required for viral suppression (34). When suppression profiles were compared across groups, we observed a delay in a subset of HIV-1C infected mice, but comparable CD4+ T cell rebound during ART across both the groups. Variations in viral suppression kinetics seen in human studies are likely due to unequal treatment free periods resulting from varied time of ART initiation and different ART regimens that do not include integrase inhibitors (31, 35). Our study utilized a three drug combination of BIC/FTC/TAF, which is the current frontline regimen recommended for treatment naïve PLHIV as well as those with advanced disease progression (36, 37). Optimal selection of ART guided by surveillance of DRM in the treatment population, would greatly enhance the probability of achieving UNAIDS 95:95:95 targets (UNAIDS, 2024). Here, we evaluated the initial viral samples from the infected mice prior to the commencement of ART for potential presence of any DRM. Our evaluation of inter subtype DRM accumulation and presence indicate disparate profiles for subtype B and C viruses during treatment naïve infection which recapitulate *in vitro* studies (38, 39). More importantly, we report a higher propensity of subtype C to accumulate DRMs specific to the regimen classes (NRTI and INSTI) used. Interestingly, a recent study reported a significant prevalence of DRMs indicative of primary resistance to NRTI and NNRTI classes of drugs in circulating virus sequenced from untreated PLHIV in India where HIV-1C predominates (40). In spite of an *in vitro* study supported ‘high genetic barrier’ (41), we observed *de novo* BIC resistant mutations in circulating viruses at study termination from the HIV-C animal that never achieved undetectable viremia following ART. Indeed, in recent preliminary reports (42, 43) virological failure was observed in PLHIV, both treatment naïve and switched to TAF/FTC/BIC regimen, within 30 months. Thus, our results need to be extended to consolidate this trend and inform drug regimens in LMICs which not only bear the highest burden of infections, but are also sites for HIV-1C dominated epidemics.

In this study, we also simulated viral rebound due to non-adherence and/or treatment interruption, a major factor associated with sub-optimal therapeutic response of ART (44–46). Viral rebound dynamics have been previously linked with pre-ART viral load, size of reservoirs expressing HIV-1 RNA in the presence of ART, and ART regimen and intermittent ‘viral blips’ (47–50). The faster rebound kinetics observed in the HIV-C group from our study is concordant with findings of a previous report in humans assessing viral rebound in HIV-1 subtype B or subtype C infected individuals receiving similar therapy (35). While the authors have attributed this property to possible suboptimal therapy adherence in the HIV-1C infected individuals due to ‘cultural and socioeconomic factors’, we believe our results, not confounded by this variable, reflect a true discriminatory inter subtype feature. As therapy adherence was controlled and monitored in our study, we believe faster viral rebound could be due to subtype C intrinsic factors such as additional NF-kb sites in the 93IN101 LTR and commonly reported for HIV-1C (51, 52) leading to faster replication and detection of rebound viruses.

Widely disseminated HIV proviral DNA, the source of active and latent reservoirs, remains the largest impediment to successful cure and eradication strategies for HIV (53). Also, as stated above, proviral load in anatomic reservoirs would be expected to influence rebound kinetics that appeared to be disparate in our study for HIV-B and HIV-C groups, albeit with a small sample size (54–56). Few human or macaque studies have addressed tissue reservoirs in the context of HIV-1C or SHIV.C. Independent studies have also reported less HIV-1 DNA in brain tissues of subtype C infected individuals compared to that in subtype B infected individuals (54, 57). Our study enabled concurrent evaluation of inter-subtype profiles of *in vivo* proviral DNA distribution in subjects undergoing an identical infection course and therapeutic regimen. We observed differential enrichment patterns in HIV-B and HIV-C groups after extended ART (58). Disparate tissue proviral distribution probably arose from the differential viral kinetics during the initial phase of the study. In the treatment interruption arm, median proviral load increase was highest for mesenteric lymph nodes in both HIV-B and HIV-C group animals, indicative of their anatomical proximity to the gut (primary site of infection) and concordant with reports demonstrating these as significant reservoir sites observed in humans (59, 60), SIV model (61) and humanized mice (24, 27). Our results also highlight bone marrow as a possible reservoir for rebound viremia, albeit to a lesser extent, for both subtypes, as has been reported in humans (62–64). Future studies using this model could now explore both active and latent viral reservoirs using viral RNA detection to inform eradication and cure strategies in a subtype specific manner.

In a previous study using humanized mice, female mice demonstrated significantly higher rates of absorption of ART drugs (65). Retrospective analysis of our data however showed that HIV-C group in spite of being populated with a greater proportion of female mice did not show faster post ART viral suppression or CD4+ T cell rebound compared to the HIV-B group. In fact, our observation seems to suggest slower response to ART, faster viral rebound upon treatment interruption and larger reservoirs for this group. These observations support a role for infecting viral subtype rather than gender to have resulted in the discernable differences seen here between the HIV-C and B groups.

The SIV/SHIV rhesus macaque model has provided critical insights into HIV transmission and pathogenesis, as well as screening for anti-retrovirals, broadly neutralizing antibodies, latency reversing agents and vaccines (66, 67). However, performing direct HIV-1 inter-subtype comparative studies described here are not possible in the NHP model. While studies using SHIV viruses employing the HIV env were informative in vaccine studies, data on important aspects of infection relating to non-env genes including accumulation and evolution of DRMs could not be obtained. Also, most SHIV.C studies have utilized HIV-1 envelopes derived from African HIV-1C strains that exhibit a distinct evolutionary signature compared to those prevalent in India, a major site for infection and evaluation for considered therapeutic strategies (68–70).

In summary, our preliminary study successfully demonstrates the utility of hu-mice in evaluating HIV-1C infection dynamics *in vivo* and comparing them with HIV-1B. Our results showed discernible differences related to replication profiles, therapeutic response and accumulation of resistance to ART and viral rebound kinetics between HIV-1 subtype B and C. However, the strong trends observed in our study need to be further confirmed with larger groups of animals.

## Data Availability

The datasets presented in this study can be found in online repositories. The names of the repository/repositories and accession number(s) can be found below: https://www.ncbi.nlm.nih.gov/, PRJNA1203808.
