# Supplementary material for: Assessing HIV-1 subtype C infection dynamics, therapeutic responses and reservoir distribution using a humanized mouse model
Source: Front Immunol. 2025 Apr 16;16:1552563. doi: 10.3389/fimmu.2025.1552563 (PMC12040690; doi:10.3389/fimmu.2025.1552563)
Supplement: Supplementary file 1 [file DataSheet1.docx]

**Supplementary Figures & Tables:**

| **Mouse ID** | **Group** | **hCD45 frequency** |
| --- | --- | --- |
| 854 | Control | 56 |
| 857 | Control | 56.5 |
| 865 | HIV-B | 52.5 |
| 869 | HIV-B | 82 |
| 871 | HIV-B | 79 |
| 875 | HIV-C | 88 |
| 891 | HIV-C | 57.6 |
| 901 | HIV-C | 74.2 |
| 904 | HIV-B | 44 |
| 906 | HIV-B | 83 |
| 907 | HIV-B | 61 |
| 909 | HIV-B | 55 |
| 912 | HIV-C | 71 |
| 917 | HIV-C | 67 |
| J224 | HIV-C | 49 |
| J225 | HIV-C | 52 |

**Supplementary table 1. Human immune cell reconstitution (hCD45+ cells) levels in study mice:** Human immune cell reconstitution was analyzed by flow cytometry at 10 to 12 weeks post-HSC engraftment. Mice with > 40% hCD45+ cells were used in these experiments.

| Purpose | Oligo name | Sequence (5’🡪3’) | Reference |
| --- | --- | --- | --- |
| Human cell number normalization | RPP38 Forward | TCACGACACCTCTGCTTTA | 24 |
|  | RPP38 Reverse | AGCGGTGAGAAACTAGGAA |  |
| Proviral DNA  (1^st^ round) | Gag1 | GATGGTGCTTCAAGCTAGTRCCAGTTGA | 25 |
|  | Gag 2 | CTCTATYTTRTCTAARGCTTCYTTGGTGTC |  |
| Proviral DNA  (2^nd^ round) | Gag 3 | CTCTCGACGCAGGACTCGGTCTGCTGA |  |
|  | Gag 4 | TTCYAGCTCCCTGCTTGCCCATACTA |  |
| gag-pol full length (DRM analysis) | F1_fw1 | CTCAATAAAGCTTGCCTTGAGTGC | 26 |
|  | F3_rev1 | GGGATGTGTACTTCTGAACTTAYTYTTGG |  |
|  | F1_fw2 | AAGTAGTGTGTGCCCGTCTGT |  |
|  | F3_rev2 | CACCTGCCATCTGTTTTCCATA |  |

**Supplementary table 2. List of primers**


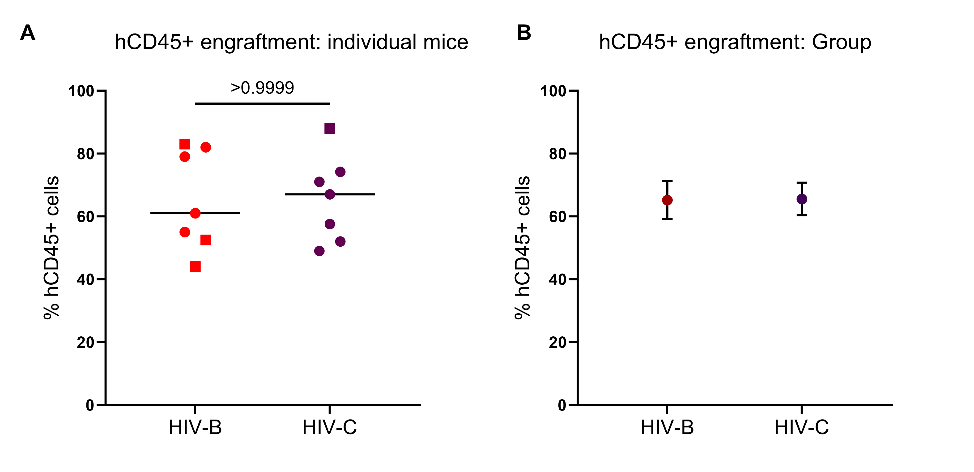


**Supplementary figure 1:** (A) Human CD45+ cell frequency at week 0 in (A) individual mice (males represented by square and females represented as circles) and (B) groupwise shown as mean ± SEM. Horizontal bars show the median of groups.


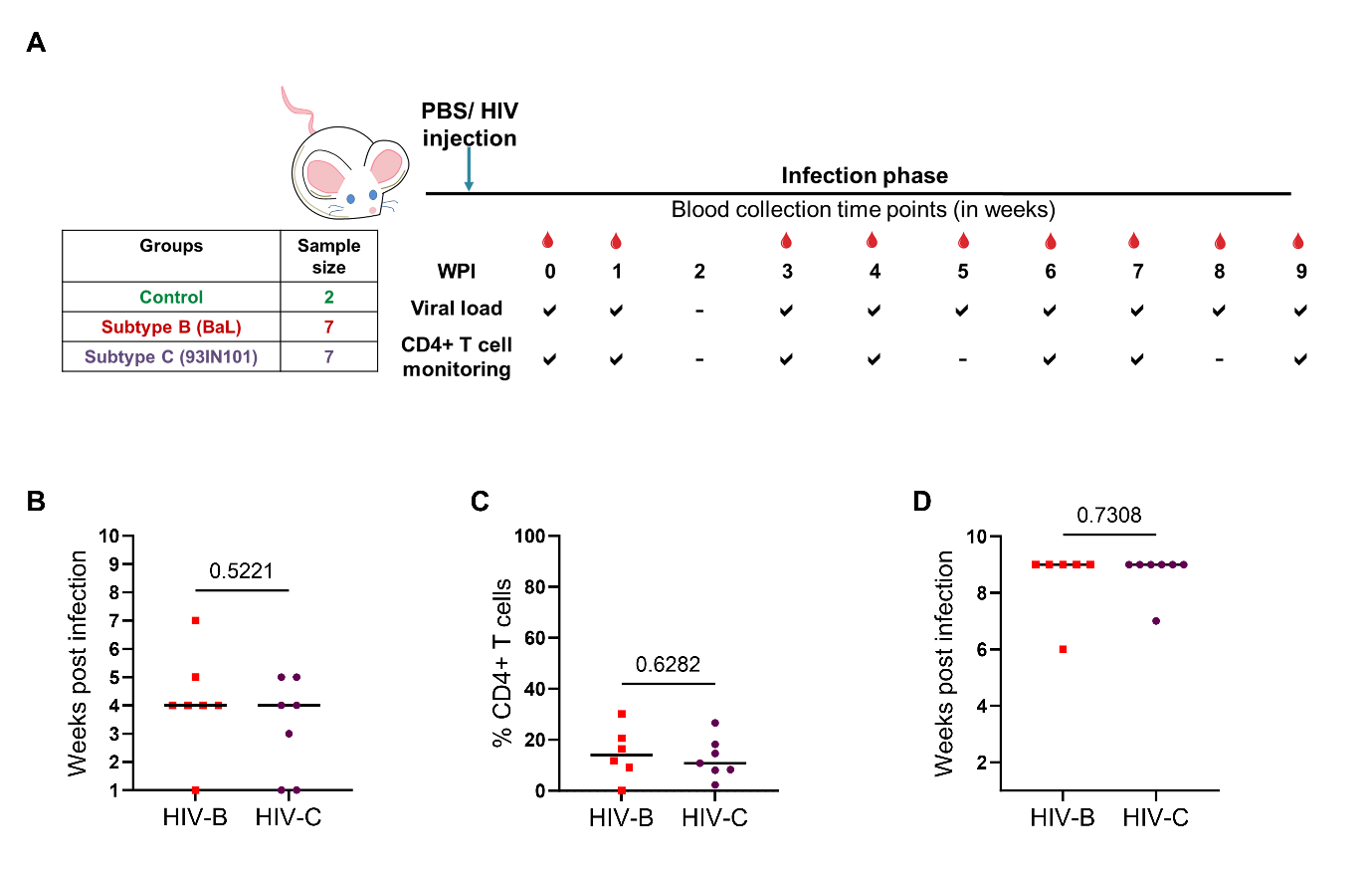
 **Supplementary Figure 2:** (A) Schematic representation of sampling during infection phase; intergroup comparison of (B) the time points (expressed as weeks post infection, WPI) at which, viremia became detectable in individual mice; (C) Nadir CD4+ T cell frequency during infection phase; (D) Time point (expressed as WPI) when lowest CD4+ T cell frequency was observed compared to the baseline i.e. 0 WPI. Statistical comparison was done using the Mann-Whitney test and p values are displayed on the plots. Horizontal bars show the median.


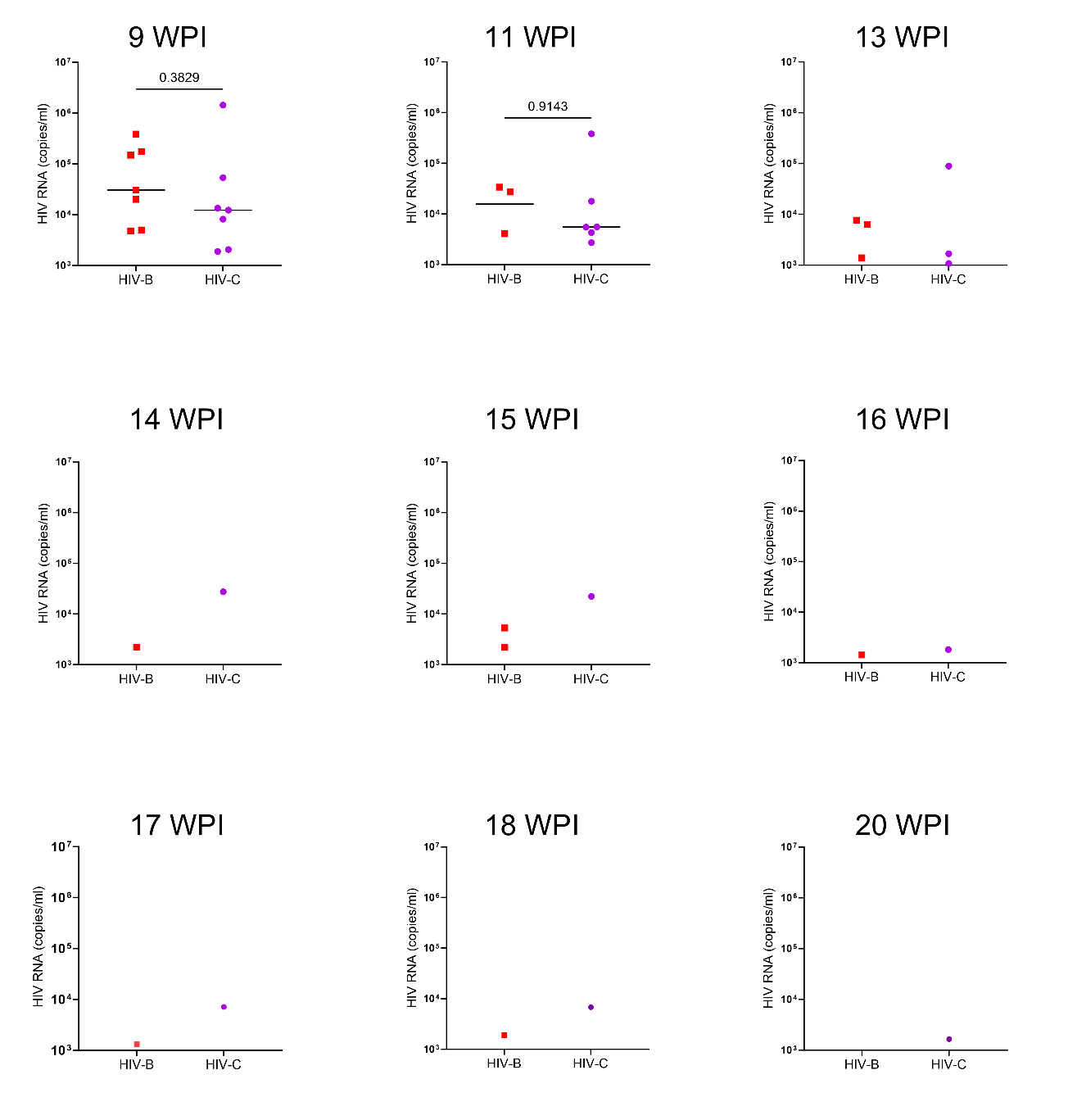


**Supplementary Figure 3. Plasma viral loads during ART phase in HIV-B & HIV-C groups**: Intergroup comparison of plasma HIV RNA copies/ml (when detectable) at various time points i.e. 9, 11, 13, 14, 15, 16, 17, 18, 20 WPI influenced by ART initiated at week 9**.** Statistical comparison was done using the Mann-Whitney test and p values are displayed on the plots. Horizontal bars show the median.


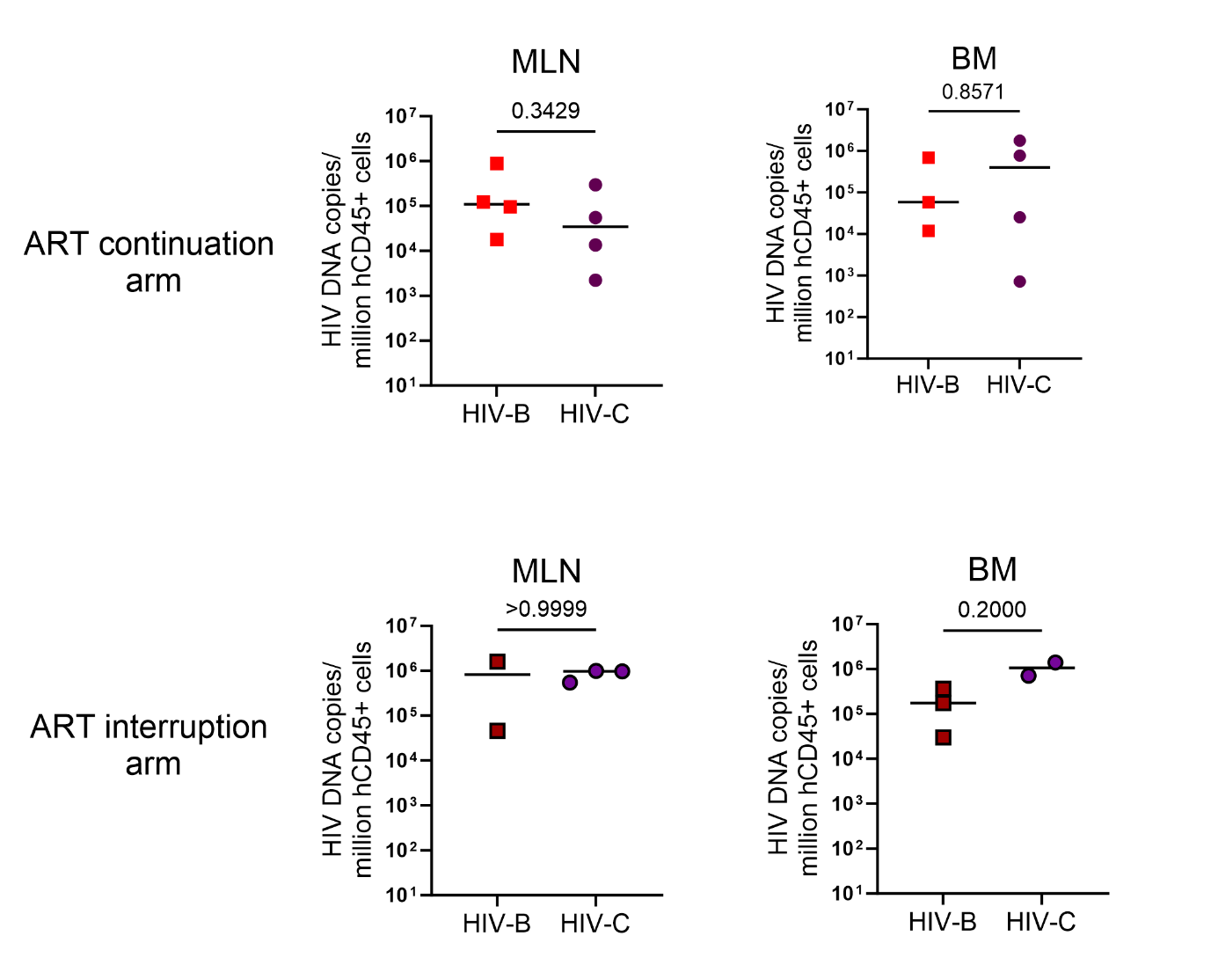


**Supplementary Figure 4. HIV proviral DNA distribution in different tissues**: Terminal tissue samples were analyzed by PCR to assess proviral loads. From left to right, HIV proviral burden in mesenteric lymph nodes (MLN) and bone marrow (BM) expressed as proviral copies per million hCD45+ cells. Symbols with bold borders indicate values for animals after treatment interruption in both the groups. HIV DNA proviral copies were compared using the Mann-Whitney test (A) ART continuation arm (B) ART interruption arm. Horizontal bars show the medians.
